# High-depth sequencing characterization of viral dynamics across tissues in fatal COVID-19 reveals compartmentalized infection

**DOI:** 10.1038/s41467-022-34256-y

**Published:** 2023-02-02

**Authors:** Erica Normandin, Melissa Rudy, Nikolaos Barkas, Stephen F. Schaffner, Zoe Levine, Robert F. Padera, Mehrtash Babadi, Shibani S. Mukerji, Daniel J. Park, Bronwyn L. MacInnis, Katherine J. Siddle, Pardis C. Sabeti, Isaac H. Solomon

**Affiliations:** 1 Broad Institute of Harvard and MIT, 415 Main Street, Cambridge, MA 02142 USA; 2 Department of Systems Biology, Harvard Medical School, Boston, MA 02115 USA; 3 Harvard Program in Biological and Biomedical Sciences, Harvard Medical School, Boston, MA 02115 USA; 4 Department of Pathology, Brigham and Women’s Hospital, Boston, MA 02115 USA; 5 Department of Neurology, Massachusetts General Hospital, Boston, MA 02114 USA; 6 Department of Immunology and Infectious Diseases, Harvard T. H. Chan School of Public Health, Harvard University, Boston, MA 02115 USA; 7 Massachusetts Consortium on Pathogen Readiness, Boston, MA 02115 USA; 8 Howard Hughes Medical Institute, Chevy Chase, MD 20815 USA

**Keywords:** SARS-CoV-2, Infection, Viral genetics

## Abstract

SARS-CoV-2 distribution and circulation dynamics are not well understood due to challenges in assessing genomic data from tissue samples. We develop experimental and computational workflows for high-depth viral sequencing and high-resolution genomic analyses from formalin-fixed, paraffin-embedded tissues and apply them to 120 specimens from six subjects with fatal COVID-19. To varying degrees, viral RNA is present in extrapulmonary tissues from all subjects. The majority of the 180 viral variants identified within subjects are unique to individual tissue samples. We find more high-frequency (>10%) minor variants in subjects with a longer disease course, with one subject harboring ten such variants, exclusively in extrapulmonary tissues. One tissue-specific high-frequency variant was a nonsynonymous mutation in the furin-cleavage site of the spike protein. Our findings suggest adaptation and/or compartmentalized infection, illuminating the basis of extrapulmonary COVID-19 symptoms and potential for viral reservoirs, and have broad utility for investigating human pathogens.

## Abstract (web summary)

Here, by high-resolution SARS-CoV-2 sequencing, genomic and transcriptomic analyses from tissue samples, Normandin *et al*. investigate viral dynamics in fatal cases of COVID-19, revealing persistent infection in distinct anatomical sites, including the heart and testis.

## Introduction

COVID-19, the most impactful global pandemic in over a century, has highlighted the ability of viruses to cause a broad spectrum of disease states with widely variable severity and symptoms. The novel coronavirus that causes COVID-19, SARS-CoV-2, is known to primarily infect lung epithelial cells^1^, yet patients frequently experience non-respiratory symptoms^2,3^, and long-term sequelae^4^.

SARS-CoV-2 infection of extra-pulmonary tissues is poorly characterized. While systemic inflammation or hypoxia could lead to the apparent disturbance of multiple organ systems^5^, it is also possible that the virus may invade and establish infection in several compartments and directly induce tissue-specific pathologies, as occurs in many other viral infections^6,7^. The putative host cell receptor (ACE2) and co-receptor (TMPRSS2)^8^ are co-expressed on many different cell types across multiple organ systems^9,10^, and in vitro experiments indicate that SARS-CoV-2 both enters and replicates in cell lines derived from several different tissues^11,12^. Studies to date have sought to identify virus across tissues, revealing systemic viral distribution in some cases^13^, and indicating several compartments that more frequently show evidence of infection, including the kidney, heart, and gastrointestinal (GI) tract^14–16^.

Molecular methods for quantifying and sequencing viral RNAs can be used to characterize viral distribution and in vivo dynamics. For example, comparing variant profiles across viral populations sequenced from different compartments may reveal tissue-specific mutations that could be involved in invasion and replication in a specific tissue or cell type, or could reflect that that tissue harbors a compartmentalized infection. Compartmentalized infection, particularly in an immune privileged site, may accommodate persistent infections, particularly in immunocompromised individuals, which fosters the generation of many novel mutations. Additionally, these sites could provide a viral reservoir that leads to reactivation or recrudescence. This phenomenon occurs across several viral families: HIV establishes a reservoir in the central nervous system^17^, flaviviruses remain in the kidneys and testis^18^, and filoviruses can also persist in the testis and other compartments^19,20^. Observations of long-lasting symptoms^21^ and apparent recrudescence in COVID-19^22^ suggest the presence of extrapulmonary reservoirs for SARS-CoV-2, although the location of these reservoirs is unknown^23^.

Investigations in natural human infections are critical to understanding SARS-CoV-2 distribution and circulation in vivo. While in vitro and animal models of infection can provide insights into many aspects of virology and host pathology, neither can fully recapitulate viral invasion and infection of multiple tissue compartments in systemic human disease^24^. While fresh or frozen human tissue is often preferred for RNA sequencing studies, the availability of such specimens outside a dedicated biobank is limited, and further complicated by biosafety considerations. In contrast, the formalin-fixed, paraffin-embedded (FFPE) specimens generated during a standard hospital autopsy for histopathological evaluation are easily stored long-term at room temperature and are non-infectious. These sample sets are underutilized in molecular assays, due to both real and perceived challenges. In particular, high-depth viral genomic sequencing can prove difficult given the high ratio of host:pathogen RNAs and potential for RNA degradation^25^.

**Fig. 1:**
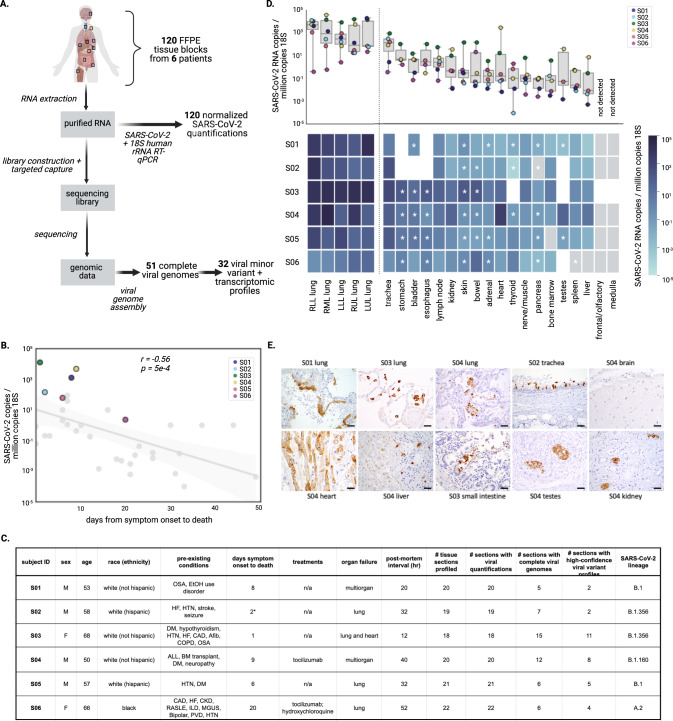
Overview of study design and sample selection. **A** Overview of sample selection and analysis of the FFPE tissue specimens (**B**) Normalized SARS-CoV-2 viral load in lung samples from a cohort of 39 subjects from which samples in the study were selected (median lung viral load is highlighted for six subjects of interest). **C** Summary of selected individuals sequenced in this study, including clinical characteristics and viral strain information. Abbreviations: OSA (obstructive sleep apnea); HF (heart failure); HTN (hypertension); DM (diabetes mellitus); CAD (coronary artery disease); COPD (chronic obstructive pulmonary disease); ALL (acute lymphocytic leukemia); BM (bone marrow); CKD (chronic kidney disease); RA/SLE (rheumatoid arthritis/systemic lupus erythematosus); ISL (interstitial lung disease); MGUS (monoclonal gammopathy of undetermined significance); PVD (peripheral vascular disease). The ‘*’ designates uncertainty around the time between symptom onset and death. **D** Boxplot (top) and heatmap (bottom) each demonstrate normalized SARS-CoV-2 quantification across tissue samples available for four or more subjects, and testis. In the boxplot, boxes delineate quartiles and whiskers show the range of all samples available (3-6 subjects). In the heatmap, composite samples (those where 2 or more tissues were in the same FFPE block) are designated with an asterisk (Supplementary Fig. 1b and Methods), gray represents virus not detected, and white designates sample was not available. **E** Representative IHC sections from study samples show the presence of SARS-CoV-2 protein (brown) in multiple tissues, including pneumocytes in lung, ciliated respiratory epithelium in the trachea, cardiomyocytes in the heart, hepatocytes in the liver, small intestine epithelial cells, rete testis, and tubular epithelium in kidneys. Staining was performed once. Scale bars are 20-microns. These findings are in agreement with sequencing data.

In this study, we outline an approach for deep profiling of viral dynamics in vivo using methods for high-depth, unbiased viral sequencing from FFPE tissue specimens and specific, sensitive identification of intrahost variants and viral transcripts. We use 120 autopsy tissue specimens from six subjects to comprehensively examine SARS-CoV-2 distribution and compartmentalization among tissues, and characterize tissue-specific variants in fatal cases of COVID-19 (Fig. 1A). We identified several extrapulmonary tissues that had high viral loads, some of which had strong genomic evidence for compartmentalized infection.

## Results

### Selection of COVID-19 autopsy tissue specimens

To elucidate viral dynamics during acute COVID-19, we identified subjects with extensive evidence of extrapulmonary pathology. This cohort was selected from 39 consecutive COVID-19 autopsies performed at Brigham and Women’s Hospital (Boston, MA) between April 14 and June 15 2020, from patients who died 0-49 days after first reported symptoms. Subjects were screened for histological evidence of COVID-19 pneumonia (i.e. diffuse alveolar damage) and presence of SARS-CoV-2 nucleocapsid antigen by immunohistochemistry (IHC). The lung IHC findings suggested that six subjects might have higher extra-pulmonary viral loads, facilitating detailed genomic studies; hence, these subjects were selected for further study (referenced as S01-S06) (Fig. 1B).

The six selected subjects all succumbed to COVID-19-related lung or multiorgan failure, with variable disease duration and virus-specific treatment regimens. They included two women and four men, were 50–68 years old, and had multiple comorbidities including diabetes mellitus (*n* = 3), hypertension (*n* = 4), and coronary artery disease (*n* = 2); one individual (S04) had acute lymphoblastic leukemia (ALL) status post bone marrow transplant (Fig. 1C). Compared to the larger cohort, the six selected subjects generally had shorter times between symptom onset and death (range: 1–20 days); previous studies^26,27^ reported that viral load is highest in specimens sampled less than two weeks after symptom onset. We also observed that lung viral load was inversely correlated with time between symptom onset and death (*r* = −0.56; *p* = 5 × 10^−4^; Fig. 1B).

For all subjects, we examined FFPE tissue specimens from the available autopsy tissue blocks from all five lung lobes (left upper lobe [LUL], left lower lobe [LLL], right upper lobe [RUL], right middle lobe [RML], right lower lobe [RLL]) and the trachea. The lungs of all subjects exhibited bilateral acute to organizing diffuse alveolar damage with edema, microvascular thrombosis, patchy bronchiolitis, and reactive epithelial changes. Trachea and bronchi exhibited reactive epithelial changes and chronic inflammation in all subjects except S02. Superimposed bacterial pneumonia involving the RML was present in S01, and LLL *Aspergillus* abscess was identified in S04.

A set of 13–17 extrapulmonary specimens per subject includes a range of extrapulmonary samples, enabling comprehensive characterization of tropism and tissue-specific viral features. This included one specimen from each of the heart, thoracic lymph nodes, kidney, liver, and spleen. Brain (frontal lobe and medulla), skin, peripheral nerve, skeletal muscle, adrenal, pancreas, thyroid, and gastrointestinal (GI) tract were examined when available. Some tissues were variably combined into single composite FFPE blocks by the clinical autopsy team and consequently analyzed together; these samples are marked with an asterisk in figures (Supplementary Data File 1, Methods). Mild chronic inflammation of the stomach, small intestine, and large intestine was identified in S03, S04, and S05. Patchy myocarditis was identified in S04.

### Quantification of SARS-CoV-2 viral load across tissues

Quantification of viral RNA by RT-qPCR enabled robust comparisons across the sample set, revealing substantial variation in viral load across different tissues. To minimize systematic variation in comparisons across subjects, tissues, and experimental batches, we normalized viral loads by specimen cellularity (reported viral load constitutes SARS-CoV-2 RNA copies per million human 18S ribosomal RNA copies) (Supplementary Fig. 1A). This normalization was particularly essential in this study given vast differences in cellularity across tissue types and specimens. Across the entire sample set, normalized viral loads ranged over eight orders of magnitude (1.1 × 10^−4^ to 3.2 × 10^4^ viral copies per million 18S) (Fig. 1D). With the exception of S03, all subjects had at least one tissue where the viral load was not detected, or detected below the assay limit of detection (Fig. 1D and Supplementary Fig. 1b).

Lung specimens generally had high viral loads relative to other tissues, while varying substantially within and across subjects. The maximum viral loads detected for each subject always occurred in a lung specimen, but loads spanned three orders of magnitude across the six subjects (1.9 × 10^1^ to 3.2 × 10^4^ viral copies per million 18S). Within subjects, the five specimens from distinct lung lobes had viral loads that varied by one to three orders of magnitude (Fig. 1D and Supplementary Fig. 1B). Trachea specimens also consistently had high normalized viral loads (highest non-lung normalized viral load in four of the six subjects).

Extrapulmonary viral load profiles across tissues were largely unique to each subject. Pancreas, liver, spleen, bone marrow, and peripheral nerve/skeletal muscle specimens were frequently associated with the lowest viral loads within subjects (Fig. 1D and Supplementary Fig. 1B). Specimens from the lymph node and GI tract had high normalized viral loads in several subjects. Other tissues, including the heart and kidney, were variable (Fig. 1D). In general, extrapulmonary viral loads were more subject-specific, as they were highest in S03 (one reported day between symptom onset and death) and S04 (immunocompromised following a bone marrow transplant). We did not observe clear trends in extrapulmonary tissue viral loads over time between symptom onset and death (Supplementary Fig. 1D).

For several tissues, high viral loads were confirmed by immunohistochemistry. Notably, the heart had the highest non-lung normalized viral load in S04 (2.06 × 10^3^ viral copies per million 18S), followed by testis and kidney (Supplementary Fig. 1B). We identified SARS-CoV-2 nucleocapsid protein in 3/6 kidney, 2/6 liver, 2/6 lymph node, 2/6 GI tract, 1/6 heart, 0/6 spleen, 0/5 brain, and 1/3 testis specimens using this method (Supplementary Data File 2). We observed SARS-CoV-2 protein in the cardiomyocytes in the heart, hepatocytes in the liver, small intestine epithelial cells, rete testis, and tubular epithelium in kidneys (Fig. 1E). To demonstrate that this signal was due to specific staining, we also stained the heart specimen where we had detected viral antigen with the nucleocapsid IHC assay (from S04), and one heart specimen where we did not (from S06), for SARS-CoV-2 spike protein, and confirmed that the results from this assay were consistent (Supplementary Fig. 1C).

### Accurate reconstruction of viral sequences from FFPE tissues

**Fig. 2:**
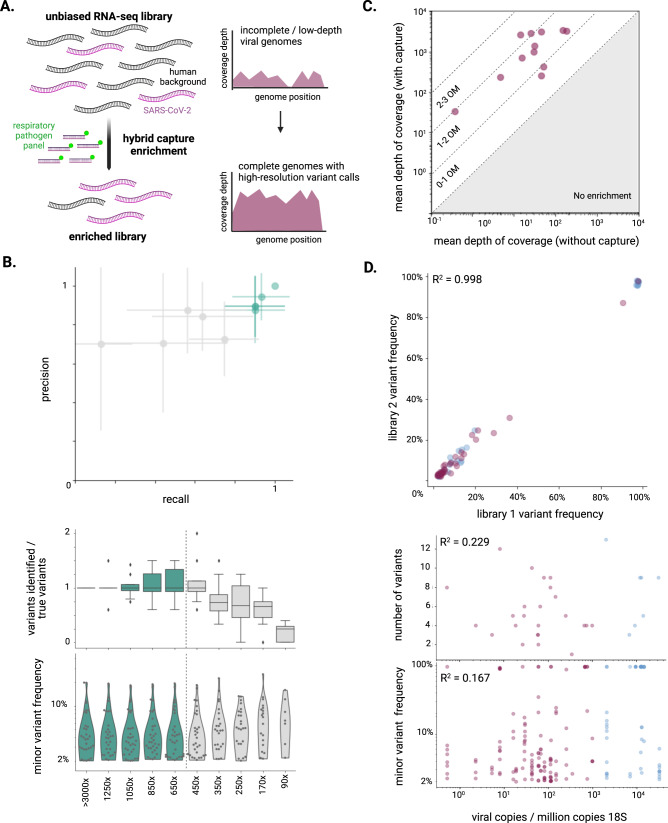
Refined sequencing methods and robust variant calling methodology enables confident analysis of variants from autopsy tissues. **A** Schematization of enhanced sequencing methods. **B** Two independent libraries from four samples with high viral depth of coverage (>3,000x mean coverage) were downsampled to a range of mean coverage depths (90x to 1,250x), and variant profiles were identified in each condition, then compared to those detected in the highest coverage condition (>3,000x mean coverage). For each library from all four samples, at each coverage depth condition, precision and recall (top; points represent the mean, while error bars represent standard deviation), the number of variants identified (middle; boxes delineate quartiles, whiskers delineate range excluding outliers), and the frequency distribution of variants (bottom; points represent variants across all samples within a condition) were compared to the highest depth of coverage condition. Green represents conditions >500x mean depth of coverage, the threshold selected for high resolution genomic analyses. **C** For 12 samples, the same libraries were sequenced with and without hybrid capture enrichment, and were then downsampled to the same number of raw reads; mean depth of viral coverage was calculated and plotted for each sample. The order of magnitude “OM” of enrichment is annotated across the two-dimensional space. **D** Variants were identified for all samples in the sample set with at least 500x mean depth of coverage, including those sequenced with hybrid capture enrichment (purple) and without (blue). Frequency of variants identified in two independent libraries were compared (top), demonstrating high correlation (*R*^2^ = 0.998). Number of variants identified (middle) and frequency of variants identified (bottom) was compared across samples, each showing poor correlation with viral load (*R*^2^ = 0.229 and *R*^2^ = 0.167, respectively).

High-depth viral sequencing proved essential for high-resolution viral genomic analyses (Fig. 2A). We sought to identify consensus-level mutations and minor variants that occurred in vivo in order to better understand circulation and compartmentalization. This aim hinged on maximizing the number of genomes assembled, and reliably characterizing their mutations. In particular, we sought to compare minor variant profiles across different tissues with highly variable viral loads without biasing identification of the number of variants or the frequencies or locations of occurrence. To explore the effect of viral load on variant identification, we serially downsampled a set of samples to different depths of viral coverage and identified minor variants (Methods). The variant profiles showed decreased recall (<0.8), or reduced sensitivity, in genomes with <500x depth of coverage (Fig. 2B top). Correspondingly, the number of identified variants was stable in samples >500x depth of coverage, but was reduced as expected at <500x depth of coverage (Fig. 2B middle). The distribution of the frequencies of minor variants was also stable in all conditions >500x depth of coverage (Fig. 2B bottom).

To maximize the number of complete viral genomes assembled from our sample set, especially those with >500x depth of coverage, we extended and validated a targeted capture approach to enable high-resolution genome analysis from tissue samples. We used a previously reported method, CATCH^28^, to design a probe set to enrich for complete genomes of SARS-CoV-2 and 20 other common respiratory pathogens (Fig. 2A). We found that targeted enrichment increased coverage depth by one to two orders of magnitude for most samples, and by more than two orders of magnitude for two samples (Fig. 2C and Supplementary Fig. 2A) Enrichment was consistent across the genome, reducing the risk of bias in variant or transcript identification (Supplementary Fig. 2B).

We applied this targeted enrichment method to our sample set and verified that variant identification was not biased by viral load. Through empirically determined normalized viral load thresholds (Supplementary Fig. 2A, Supplementary Data File 3, Methods), we identified 74 total samples to sequence and 58 to enrich through hybrid capture. From these samples, we were able to assemble complete SARS-CoV-2 genomes (>90%) from 51 samples, 32 of which had mean depth of coverage >500x (Supplementary Data File 4). In samples with >500x depth of coverage, variant frequencies were consistent across two independently generated libraries (R^2^ = 0.998), indicating that the variant identification was robust (Fig. 2D top). As expected, the number of variants identified did not correlate with viral load (R^2^ = 0.229) or variant frequency (R^2^ = 0.167) (Fig. 2D). As in the head-to-head comparison described above, samples sequenced with and without targeted enrichment exhibited a similar distribution of coverage across the genome (Supplementary Fig. 2C); additionally, FFPE samples behaved similarly to frozen samples with similar viral loads (Supplementary Fig. 2D).

### Comparison of viral genomes and transcripts across samples

**Fig. 3:**
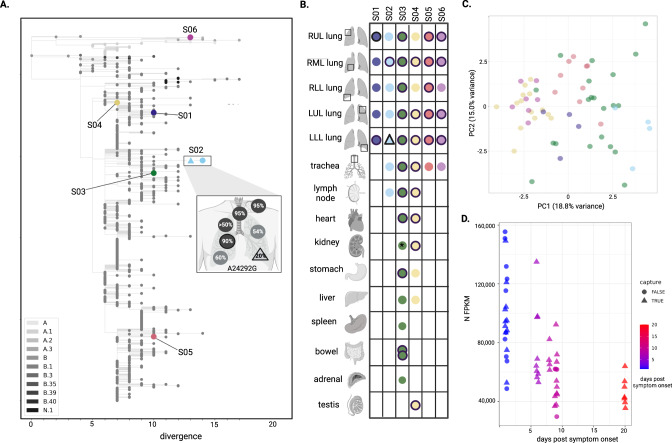
Complete SARS-CoV-2 genomes across subjects and tissues. **A** A maximum likelihood phylogenetic tree of the unique viral genomes identified, including two unique consensus genomes from S02, demonstrates genetic divergence. The viral sequences are presented in the context of 729 sequences from a six month window centered on the time these infections occurred, with color representing SARS-CoV-2 Pango lineage (legend). Inset, the SNP that differentiates unique genomes from S02 (A24292G) is highlighted; allele frequencies across all seven tissues from which a genome assembled are annotated (for two samples, black outline designates >500x mean depth coverage for high-confidence variant quantification). **B** A schematic representation of complete viral genomes assembled across the six subjects. Colored shapes represent the assembly of a complete genome, each unique color-shape combination represents a unique genome, which is represented in the phylogenetic tree. A black outline designates >500x mean depth coverage, suitable for minor variant and transcriptomic analysis. **C** Principal component analysis of viral fragment abundance for 32 unique samples (those with >500x mean depth of viral coverage, some being duplicate libraries), colored subject, demonstrate that samples separate in PC1 by time between symptom onset and death. **D** Nucleocapsid (N) gene expression, normalized by the total number of viral reads, decreased with time between symptom onset and death. Legends indicate day between symptom onset and death, and whether a sample underwent targeted enrichment.

Analysis of the 51 complete SARS-CoV-2 consensus genomes from different tissues and subjects revealed significant inter-subject diversity but limited intra-subject diversity. Genomes from the six subjects were well-distributed among 729 genomes sampled in the Northeast US from January - June 2020, and differed from each other by five to 27 single nucleotide polymorphisms (SNPs) (Fig. 3A and Supplementary Fig. 3A). This analysis demonstrates that all subjects were infected with the B.2 strain of SARS-CoV-2, with the exception of Subject 6 who was infected with the ancestral A.2 lineage (Figs. 3A and 1C). For each subject, we had genomes from all five lung lobes as well as extra-pulmonary tissues from S02, S03, and S04 (one, nine, and six additional tissues, respectively) (Fig. 3B). All consensus-level genomes within subjects were identical, with one exception; the LLL lung from S02 had the ancestral allele (A) at position 24,292, while in all other tissues, the dominant allele at that site was a synonymous mutation (A24292G) (Fig. 3A, B). The A24292G mutation was still present in the LLL sample but at 20% frequency. Similarly, there was a mix of the ancestral and mutant allele in other samples from this subject (LUL, RUL, RML, RLL, trachea, and lymph node) (Fig. 3A). Since A24292G was not common in strains circulating at the time, we hypothesize that this mutation arose in the LLL, then achieved higher frequency in other compartments through bottlenecking during spread.

In order to better understand subject- or tissue-specific viral activity, we directly quantified per-sample viral transcript abundance and performed principal component (PC) analysis on the normalized transcript abundance matrix (Fig. 3C). The first PC accounted for 19% of the total variance and was anti-correlated (spearman rho = −0.81) with subject of origin and time post symptom onset. Examination of the PC1 loadings revealed that the sample separation along this component was primarily driven by viral regions ORF1ab, M and ORF10, collectively accounting for more than 50% of the loading. We used pairwise correlation to examine the relationship in viral RNA abundance levels between different viral genes. There was strong anticorrelation (rho = < −0.74) of ORF1ab with the ORF10, N, E and M genes and to a much lesser extent with other viral genes (Supplementary Fig. 3B). The abundance level of four genes (ORF1ab, E, M, N, ORF10) was correlated (|rho | > 0.5) with date post symptom onset indicating that the gene anticorrelation observed was directly related to time post-symptom onset (Fig. 3D and Supplementary Fig. 3C). This observation suggests that RNA viral abundance of the N, E, M and orf10 viral genes could be specifically reduced during late stages of infection.

To further investigate the cause of these expression changes, we developed and applied both an RT-qPCR assay and the novel Antenna computational pipeline and used it to quantify subgenomic RNA (sgRNA) levels (Methods). Viral sgRNAs are generated in infected cells where the virus is replicating, and can indicate viral activity^29^. The sequencing data did not show monotonic trends in sgRNA abundance (with the exception of the S gene sgRNA, which decreased as the number of days post symptom onset increased) (Supplementary Fig. 3D), suggesting that this apparent difference in abundances of gene sequences cannot be explained by sgRNA levels. To validate this result, we also developed an RT-qPCR assay targeting subgenomic nucleocapsid (sgN) RNA, then applied it to the sample set, revealing sgRNAs in many samples (Supplementary Data File 1). The sgN quantification was highly correlated with total viral RNA quantification; sgN was present at an average proportion of 11% of total N RNA (Supplementary Fig. 3E). There were no clear tissue-specific trends in the proportion of sgN across or within subjects (Supplementary Fig. 3F). Comparison of sequencing and qPCR quantification showed good agreement for the N gene between techniques (p-value = 3.53 × 10^−5^; Supplementary Fig. 3G).

### Comparison of viral genomic features within subjects

We next sought to use minor variants to uncover viral evolution and circulation among tissues. Minor variant profiles were analyzed in the 32 samples that passed the stringent threshold of >500x mean viral depth of coverage. These included five extrapulmonary samples from S03, and four from S04. Out of the 136 total variable positions in this sample set, only five positions were common between subjects (Supplementary Fig. 4A). Overall, positions of variation were well distributed across the genome, and 58% of variants were nonsynonymous, consistent with previous studies^30,31^. Most variants (88%) were low frequency (defined as <10% or >90% frequency).

**Fig. 4:**
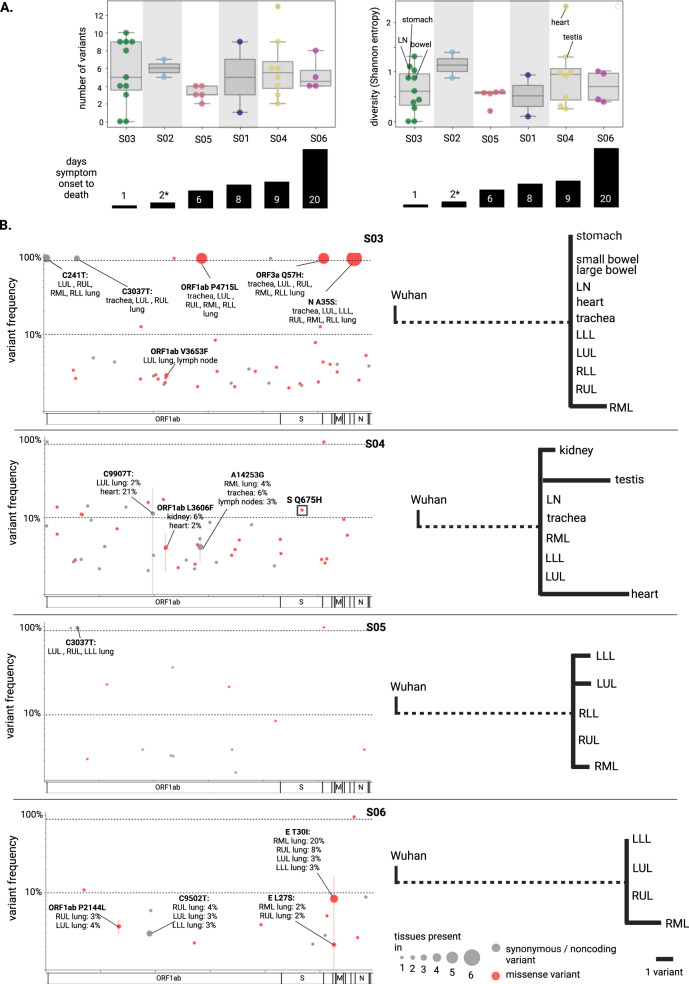
SARS-CoV-2 minor variants across tissues. **A** Number of variants and viral population diversity (Shannon entropy) for each sample with >500x depth of coverage, arranged by subject (ordered by time between symptom onset and death). In the boxplot, boxes delineate quartiles and whiskers show the range, excluding outliers (as determined by the interquartile range). S01 and S02 had only two samples for comparison and were not analyzed further for circulation or compartmentalization; the remainder of the subjects had 4–11 samples. The ‘*’ designates uncertainty around the time between symptom onset and death. **B** On the left, all variants are displayed as genome position vs frequency for each of subjects S03-S06. The size of the points reflects the number of tissues the variant was observed in; the standard deviation of the frequency at which the variant occurred across tissues is depicted by error bars. Red points reflect nonsynonymous changes whereas grey points reflect synonymous or noncoding variants. Dashed lines are present at 10 and 90% frequency; variants falling between dashed lines were considered high-frequency. On the right, high-frequency variants were quantified and diagrams were constructed to demonstrate genetic distance. Dashed line represents the number of SNPs differentiating the consensus genome from SARS-CoV-2 Wuhan reference strain (NC_045512.2).

The viral population diversity, as determined by the number of minor variants and the frequency at which they occur, varied across subjects and tissues. The number of variants identified in each sample ranged from zero to 13 (Fig. 4A). We observed no minor variants in two tissues from S03, the subject with the shortest course of disease. We observed the most variants and the highest diversity in the heart sample for S04 (Shannon entropy = 2.3); this was an outlier when compared to the mean value for that subject (Shannon entropy = 0.94). The testis sample also had high diversity.

We specifically compared variant profiles within subjects, finding few minor variants that were shared among tissues within subjects, particularly later in infection. We focused on S03-S06, since we had high-depth genome coverage from at least four tissues for these subjects. The patterns of variant sharing can illuminate paths of circulation or lack thereof. Several variants were shared among respiratory tract samples from S03, and one variant was shared between lung samples in S05 (Supplementary Fig. 4B); both of these subjects had a relatively shorter course of disease (one and six days, respectively). We would expect variant frequency to be concordant across tissues given perfect circulation, whereas the presence of high-frequency, tissue-specific variants indicates a lack of circulation, i.e. compartmentalization. In S04 and S06, two subjects with a longer disease course (nine and 20 days, respectively), the variants that were shared among tissues occurred at notably different frequencies across tissues (e.g. the E T30I mutation was at 3% in the LLL but 20% in the RML of S06, and C9907T was at 2% in the LUL but 21% in the heart of S04), suggesting increased compartmentalization (Fig. 4B).

We focused on tissue-specific high-frequency variants to systematically assess compartmentalization within subjects, revealing strong evidence of extrapulmonary compartmentalization in one subject. S03, S05, and S06 each had tissues with one to two high-frequency variants. Strikingly, S04 had nine high-frequency variants: four were in the testis and five were in the heart (Fig. 4B). These high-frequency variants were consistently detected in two independently constructed libraries, and they were undetectable in two independently constructed libraries from all other tissues from this subject (with the exception of C9907T, which was detected at 2% in the LUL, as noted above) (Supplementary Fig. 4C). These two tissues were divergent from other tissues from this subject, indicating either compartmentalization, or an adaptation for invading or replicating in that tissue. We investigated whether these variants have been observed in other studies; GISAID data revealed that one mutation, Spike (S) Q675H, has previously been observed, present at as much as 3% frequency worldwide (January 2021). This mutation was not circulating at the time that this subject contracted SARS-CoV-2, but has since emerged in multiple SARS-CoV-2 lineages, indicating convergent evolution^32,33^. This mutation falls directly upstream of the furin-cleavage site and is predicted to enhance viral entry^32,33^. This suggests that this was a functionally advantageous mutation for either infecting the heart specifically, or it may have been broadly advantageous, but did not spread to other tissues because the infection was compartmentalized.

## Discussion

In this study, we used molecular tools to characterize the anatomical distribution of SARS-CoV-2 and investigate its evolution and circulation during natural human infection. By applying multiple RT-qPCR assays, high-depth sequencing, and fine resolution genomic analyses, we were able to shed light on the basis of tissue-specific pathologies, observe trends that differentiated patients with shorter and longer disease courses, and identify extrapulmonary tissues that harbor compartmentalized infection later in disease.

This study was made possible by our development and validation of enhanced methods to sequence viral genomes from FFPE tissue specimens. Viral sequencing and genomic analyses from tissues present additional challenges over fluid samples (e.g. blood, urine, swabs), particularly high host RNA background, but enrichment for viral sequence through hybrid capture enabled high-depth genome sequencing. Despite the chemical crosslinking in FFPE samples, we found that viral genome coverage obtained from FFPE samples was comparable to that from frozen tissues. Our analysis shows that minor variant identification sensitivity is consistently reliable above 500x mean depth of coverage, but sensitivity drops considerably at lower coverage depths. While this is notable for any study of intrahost variation, it is particularly vital to keep in mind for studies comparing variant profiles across samples with vastly different viral loads and corresponding coverage depths. We demonstrated the utility and practical relevance of this method by deploying it to assemble viral genomes from dozens of tissue samples from six fatal cases of COVID-19, the majority of which had >500x coverage depth. There was very little SARS-CoV-2 variation in consensus-level genome assemblies between tissues in the same indivudual, underscoring the importance of accurate minor variant calling to characterize in vivo evolution.

We identified viral genomic and transcriptomic commonalities and differences between subjects, and found indications of latency and compartmentalization that were particularly associated with longer infection times. Although we observed heterogeneity in the tissues affected, each subject had relatively high viral loads in several extrapulmonary tissues, and viral loads were generally lower in subjects with a longer disease course. We found that N gene RNA abundance, but not the proportion of N gene sgRNA reads, also decreased with a longer time interval post symptom onset. This suggests a down-regulation of viral structural components specifically later in the disease course, which could play a role in immune evasion. Viral suppression of short sgRNA expression has been demonstrated in the context of SARS-CoV-1^34^, as has regulation of subgenomic RNA synthesis^35^. These findings raise the possibility that SARS-CoV-2 enters a slower replication state as infection continues, and warrants further investigation in larger cohorts. We also identified more evidence of compartmentalization in subjects with longer times between symptom onset and death, with S03 and S04 providing a striking comparison point. S03 had no tissue-specific high-frequency variants in any of the five extrapulmonary tissues profiled. This subject had only one day between symptom onset and death, and the short disease course, likely not providing sufficient time for compartmentalization to establish. By contrast, S04, with nine days between symptom onset and death, had 10 tissue-specific high-frequency variants, all in extrapulmonary tissues, strongly suggestive of compartmentalization.

This evidence for compartmentalized infection, particularly in an immune-privileged site, has ramifications for SARS-CoV-2 evolution, as well as COVID-19 diagnostics and treatment. Compartmentalized infection in the heart of S04 was consistent with the very high viral load by RT-qPCR and strong histopathological evidence for infection of the cardiomyocytes in this subject, as well as findings from previous reports of SARS-CoV-2 in the heart^36^. This lends credence to the hypothesis that direct infection may lead to the heart-related sequelae observed in some COVID-19 patients. The testis, which also had evidence of compartmentalized infection in S04, is a known immune-privileged site and has been demonstrated to harbor compartmentalized infections of various pathogens^37–39^, with implications for fertility and sexual transmission^40^. SARS-CoV-2 may establish a viral reservoir in these anatomical sites, leading to ongoing tissue-specific pathology, and/or eventual reactivation. Moreover, a persistent infection, particularly in an immunosuppressed patient, provides an environment for sustained viral evolution, which can result in the emergence of variants within the infected host^22,41^, and has been hypothesized as a potential source for novel variants of concern^22,41^. The Spike Q675H mutation, an adaptive mutation that has emerged independently in several SARS-CoV-2 lineages^32,33^, arose in a compartmentalized infection in one individual (who was immunocompromised) after just 9 days of infection. The emergence of a functionally advantageous mutation in the heart in a short period of time highlights the potential role of extra-pulmonary reservoirs of SARS-CoV-2 in viral evolution within persistently infected hosts.

While many of our findings warrant further investigation, we acknowledge several limitations of this study, especially in the size of sample set and cohort. The cohort consisted of six subjects with diverse clinical histories, symptoms, and treatment courses, confounding direct comparisons across subjects. Given that all subjects had considerable comorbidities, and ultimately succumbed to fatal disease, we cannot generalize about the dynamics of SARS-CoV-2 in all patients with COVID-19. Autopsy post-mortem intervals also varied across subjects, which may have resulted in differential RNA quality between subjects. Additionally, these subjects were sampled from a brief period at the beginning of the pandemic in a single geographic region, and the diversity of SARS-CoV-2 genomes at the time was limited, while there were also genetic differences among infecting strains that are not well-characterized. Importantly, all of our subjects had relatively short courses of disease, in comparison to reports of persistent infections^22,42–44^. We would expect stronger evidence of compartmentalized infection in more prolonged infections, but in this study we did not identify any such subjects with evidence of high enough viral loads to support high-depth sequencing.

We have detailed enhancements in viral sequencing that could enable high-resolution, fine-scale viral genomic analyses in future studies focused on SARS-CoV-2 variants, other coronaviruses, or a myriad of other viral families, especially those that are known or thought to establish reservoirs during infection. Given the abundance of FFPE specimens representing broad sets of tissues from humans with diverse disease states collected over many years, it is our hope that the approach we describe here will offer great potential to produce further insights into basic virology and the development of therapeutic strategies.

## Methods

### Autopsies and Tissue collection

This study was approved by the Mass General Brigham Institutional Review Board under a protocol allowing for use of excess tissue not required for diagnosis that was collected during routine hospital autopsy examination (#2015P001388). The protocol waived the requirement for consent from subjects who participated in the study due to their deceased status and overall risk, which was deemed minimal. Consent for the hospital autopsy was previously given by the decedents’ next of kin or health care proxy per Massachusetts state law, with agreement that tissue retained by BWH could be used for IRB-approved research studies. Patients with autopsies performed at Brigham and Women’s Hospital were included if a history of SARS-CoV-2 infection was confirmed by pre- or perimortem nasopharyngeal swab RT-qPCR or serology. Eviscerations were performed in a negative pressure isolation suite with personnel equipped with N95 or powered air purifying respirator (PARP) masks, and dissection of organs in a biosafety hood. Representative tissue sections from lungs, trachea/bronchi, heart, liver, kidneys, spleen, large intestine, small intestine, thyroid, pancreas, adrenals, bladder, uterus, ovaries, testis, skin, skeletal muscle, peripheral nerve, and brain were fixed in 10% formalin and processed by standard histology protocols prior to paraffin embedding. SARS-CoV-2 nucleocapsid immunohistochemistry was performed as previously described^45^. Additionally, IHC for the SARS-CoV-2 spike protein was performed using a mouse monoclonal antibody (GTX632604; GeneTex, Irving, CA; 1:1000 dilution). Clinical history was extracted from the electronic medical record.

### RNA extraction and purification

For each sample analyzed (Supplementary Data File 1), we extracted three 20-μm scrolls of FFPE tissue using a DNA/RNA FFPE miniprep kit (Zymo Research), with water extracted alongside each batch to serve as a negative control, as previously described^37^. DNA was depleted from nucleic acid samples using Turbo DNAse (Thermo Fisher Scientific), and RNA was purified using AMPure XP beads (Beckman Coulter), eluted in 15 μL of water.

### SARS-CoV-2 RT-qPCR RNA quantification

We used an RT-qPCR assay targeting the nucleocapsid gene, based on the CDC N1 SARS-CoV-2 RNA detection assay, to quantify viral RNA, as described^46^. We performed the assay on 1 μL of purified RNA samples (diluted 1:3) per 10 μL reaction in triplicate, alongside extraction water controls, in-assay negative controls, and a synthetic dsDNA standard. Using the in-assay standard curve, we calculated viral load, correcting for the sample dilution factor. We used 18S ribosomal RNA as a housekeeping gene^47^ to quantify cellular content in these samples. We performed a previously published RT-qPCR assay targeting this region^48^ on 1 μL of purified RNA from each sample (diluted 1:100) in triplicate, alongside in-assay negative controls and a synthetic dsDNA standard. RT-qPCR data was generated using Quant Studio 6 Flex Real-Time PCR System Software

### SARS-CoV-2 RT-qPCR subgenomic quantification

We developed a RT-qPCR assay targeting the subgenomic RNA for the nucleocapsid. We adapted the method described by Wolfel et al.^29^ to design a forward primer targeting the common leader sequence (5’-CGATCTCTTGTAGATCTGTTCTC-3’) shared by all subgenomic RNAs (sgRNAs). When combined with the reverse primer from the N1 assay, we were able to quantify the subgenomic fragments encoding the nucleocapsid protein (sgN). We used this primer set to amplify sgN from viral RNA, confirmed that the amplicon was the expected size by gel electrophoresis, and verified that it was the expected sequence by Sanger sequencing. In order to validate our assay, we designed a synthetic DNA fragment containing the leader sequence, a linker, and the N’ terminus of the N1 gene. This synthetic fragment is amplified by both N1 primers and sgN primers, allowing us to create comparable in-assay standard curves and control for differences in amplification efficiency. RT-qPCR quantification of sgN was performed in the same manner as N1 with equivalent reagents and cycling conditions. This assay was applied to a subset of samples (Supplementary Data File 1).

### RNA sequencing library construction

For all samples sequenced, we first depleted ribosomal RNA from purified RNA using an RNAse H-based approach described^49^. We then performed ligation-based cDNA synthesis and library construction using a TruSeq stranded total RNA kit (Illumina) with a 1 minute fragmentation time and with 0.2 μM xGen UDI-UMI adapters (IDT). Libraries were quantified with TapeStation high-sensitivity DNA assay (Agilent).

### Hybrid capture SARS-CoV-2 enrichment

We implemented CATCH^28^ to design a set of probes that could be used to enrich for SARS-CoV-2 complete genomes. This V-Respiratory probe set contains 100,000 probes that cover all known genome diversity of a panel of 20 respiratory viruses using sequencing data compiled as of 02/18/2020^50^. The panel was ordered from Twist as a Custom Panel (Twist Bioscience). With this probe set, we performed hybrid capture using the Twist Hybridization and Wash Kit (Twist Bioscience), according to the Twist Target Enrichment Protocol Appendix Y. Samples that underwent hybrid capture were combined in equimolar amounts in pools of up to 12 samples (with distinct sequencing indices).

### Sample set sequencing

We performed library construction and hybrid capture on a limited set of 44 samples with varied viral loads to determine which samples were likely to yield a genome, and which would benefit from enrichment through hybrid capture (Supplementary Data File 3). From these data, we determined that sequencing samples with less than 0.1 SARS-CoV-2 / million copies 18S was unlikely to yield a complete genome, and samples with less than 1,000 SARS-CoV-2 / million copies 18S (Supplementary Fig. 2A) would require hybrid capture to recover sufficient genome coverage for genome assembly and confident minor variant calling; we thus set normalized viral load thresholds for which samples to sequence, and which to subject to hybrid capture. Duplicate libraries were attempted for all samples where an initial library had yielded a genome. After library construction, with or without additional hybrid capture, samples were pooled at equimolar ratios and sequenced on a NovaSeq SP (Illumina) with 2x146bp cycles.

### Viral genomic and transcriptomic analyses

Viral genomic analyses were performed through use of viral-ngs pipelines (dockstore.org/organizations/BroadInstitute/collections/pgs), as implemented on Terra platform (app.terra.bio). Samples were demultiplexed using the demux_only pipeline with read_structure=146T8B9M8B146T. External sequencing data for comparisons to frozen samples were obtained from NCBI BioProject PRJNA720544, generated in a prior study^51^.

#### Downsampling

For serial downsampling used to benchmark viral variant calling by coverage depth (Fig. 2B), mapped viral read bam files were downsampled using the downsample workflow to within 10% of the reported mean depth of viral coverage. For head-to-head comparison of libraries sequenced with and without hybrid capture, all samples were downsampled to 3 million raw reads (without prior deduplication) (Fig. 2C).

#### SARS-CoV-2 consensus genome assembly and analysis

SARS-CoV-2 genomes were assembled using the assemble_refbased workflow (viral-ngs version 2.0.21), using the SARS-CoV-2 reference NC_045512.2. A selection of standard outputs from this workflow were reported in Supplementary Data File 4. Genomes with >90% unambiguous base pairs were considered complete. All complete genomes derived from the same subject were aligned to each other using MUSCLE^52^, as implemented in Geneious Prime (v2021.1.1). Unique consensus genomes from the six subjects were also aligned to each other, and distance matrix was reported (Supplementary Fig. 3A). Viral strains classification was performed by search against using the Usher web interface on 09-21-2021 against the full database.

#### Viral variant identification and analysis

On all genomes with >500x mean depth of coverage, we used LoFreq (with parameters -q 20 and -Q 20) to identify variants, relative to the SARS-CoV-2 reference sequence (NC_045512.2)^53^. We filtered out variants identified that were <2% frequency or >98% frequency (relative to reference), as well as those at sites with depth of coverage <100 and variant reads <5. We used SnpEff to annotate variants^54^, and subsequent analyses of variant positions, variant type, and variant location were derived from this annotation. BWH_189, a kidney section from S03, had high-frequency minor variants that matched consensus-level SNPs from another subject in our cohort. This indicated contamination during sequencing, and thus this sample was removed from analyses. In cases where each replicate yielded a genome with >500x depth of coverage, variants from each duplicate library were combined across replicates. We used separate minor variant profiles from each replicate to validate minor variants of interest in S04. In relevant cases, duplicate libraries were merged in an attempt to assemble a complete genome or to produce a genome with >500x depth of coverage. Details about where duplicate genomes and minor variant profiles were available, as well as cases where sequencing data was merged, are available in Supplementary Data File 4. To calculate the viral population diversity using Shannon entropy for each sample: we took the negative sum of the product of the frequency multiplied by the natural logarithm of the frequency of each variant.

#### Computational viral gene and sgRNA quantification

Viral gene quantification was performed using featureCounts command of the subread package (version 2.0.1). Gene annotation was derived from the RefSeq NC_045512 record using BioPython (1.79)^55^. sgRNA quantification was performed using the custom antenna pipeline. The Antenna pipeline utilizes local alignment of soft-clipped virally aligned reads to identify TRS containing sequences^56^ in next-generation sequencing (NGS) data. Briefly, reads were converted to fastq format and aligned to the viral genome using BWA including the -Y flag to include soft-clipped portions of reads. Reads were processed using a custom python script that identified transcription-regulating sequences (TRS) sequences in the softclipped regions, performed local alignment of 3’ and 5’ soft-clipped sequences against all orientations of the TRS sequence and quantified reads in a read pair aware manner. Cutoff for the identification of TRS containing reads was set to 30 after manual inspection of the distribution of scores for all read orientations. Three of the samples with the highest number of reads (BWH101_1, BWH160_2, BWH165_1) were downsampled to 30% of the input reads prior to sgRNA NGS quantification due to computational constraints, one sample (BWH158) was downsampled to 10% of the original number of reads. These analyses were performed on samples with high depth viral sequencing, and notably we omitted samples from S02, where there was uncertainty about time between symptom onset and death. The Antenna pipeline is available at https://github.com/broadinstitute/antenna^57^.

#### Phylogenetic analysis

In order to construct a phylogenetic tree, consensus viral genome sequences, generated as described above, were aligned to the reference SARS-CoV-2 sequence using the mafft software (version 7.487), with parameters --addfragments and --keeplength. We constructed a phylogenetic tree using the sarscov2_nextstrain_aligned_input pipeline in viral-ngs. For the six subjects in our cohort, precise collection dates were not available, so dates were randomly assigned within a 2 week window. We provided the above pre-aligned sequences as input, required their inclusion in the output tree and furthermore added 729 contextual sequences within a six-month window centered on the sample collection dates. Tree graphics were generated using the baltic python package^58^.

### Statistics and Reproducibility

No statistical method was used to predetermine cohort sample size. Subjects were a convenience sample composed of individuals who had an autopsy performed at BWH following SARS-CoV-2 infection; blinding and randomization were not relevant to this study. Each FFPE tissue block was stained once with the respective SARS-CoV-2 nucleocapsid or spike antibodies; these antibodies have been extensively validated for diagnostic use in our clinical IHC laboratory, including reproducibility between staining batches. RT-qPCR experiments were performed in triplicate; mean quantifications are reported. Sequencing was performed and analyzed in duplicate, where possible. Variant profiles from samples with low mean viral coverage (<500x) were excluded from analyses, consistent with the threshold determined in this study (Fig. 2).

### Reporting summary

Further information on research design is available in the Nature Research Reporting Summary linked to this article.

## Supplementary information

Supplementary Information

Description of Additional Supplementary Files

Supplementary Dataset 1

Supplementary Dataset 2

Supplementary Dataset 3

Supplementary Dataset 4

Supplementary Dataset 5

Reporting Summary

## Data Availability

The sequencing data generated in this study have been deposited on NCBI under the accession code PRJNA720544. Unique SARS-CoV-2 consensus genomes from this study are deposited on GenBank under accessions OP607135-OP607141. Other data are available upon request. Source data are provided with this paper.

## Source data

Source Data

## Code availability

All genomic analysis pipelines are publicly available, as described throughout the Methods. Figures were made using R, Python, and BioRender.com. The Antenna Pipeline is publicly available at https://github.com/broadinstitute/antenna^57^.
