# Supplementary material for: High-depth sequencing characterization of viral dynamics across tissues in fatal COVID-19 reveals compartmentalized infection
Source: Nat Commun. 2023 Feb 2;14:574. doi: 10.1038/s41467-022-34256-y (PMC9894515; doi:10.1038/s41467-022-34256-y)
Supplement: Supplementary file 1 — Supplementary Information [file 41467_2022_34256_MOESM1_ESM.pdf]

Title

High-depth sequencing characterization of viral dynamics across tissues in fatal COVID-19 reveals compartmentalized infection

Author list

Erica Normandin[1,2]*, Melissa Rudy[1], Nikolaos Barkas[1], Stephen F. Schaffner[1], Zoe Levine[1,3], Robert F. Padera, Jr.[4], Mehrtash Babadi[1], Shibani S. Mukerji[5], Daniel J. Park[1], Bronwyn L. MacInnis[1,6,7], Katherine J. Siddle[1,2]*, Pardis C. Sabeti[1,6,7,8], Isaac H. Solomon[4]*.

Affiliations

[1] Broad Institute of Harvard and MIT, 415 Main Street, Cambridge, MA 02142, USA, [2] Department of Systems Biology, Harvard Medical School, Boston, MA, USA, 02115, [3] Harvard Program in Biological and Biomedical Sciences, Harvard Medical School, Boston, MA, USA, 02115, [4] Department of Pathology, Brigham and Women's Hospital, Boston, MA 02115, [5] Department of Neurology, Massachusetts General Hospital, Boston, MA 02114, [6] Department of Immunology and Infectious Diseases, Harvard T. H. Chan School of Public Health, Harvard University, Boston, MA 02115, USA, [7] Massachusetts Consortium on Pathogen Readiness, Boston, MA 02115, USA, [8] Howard Hughes Medical Institute, Chevy Chase, MD, 20815.

These authors jointly supervised this work: Katherine J. Siddle, Pardis C. Sabeti, Isaac H. Solomon

* Corresponding authors

Corresponding author emails: erica.normandin@g.harvard.edu, kjsiddle@broadinstitute.org, ihsolomon@bwh.harvard.edu

Supplementary Information

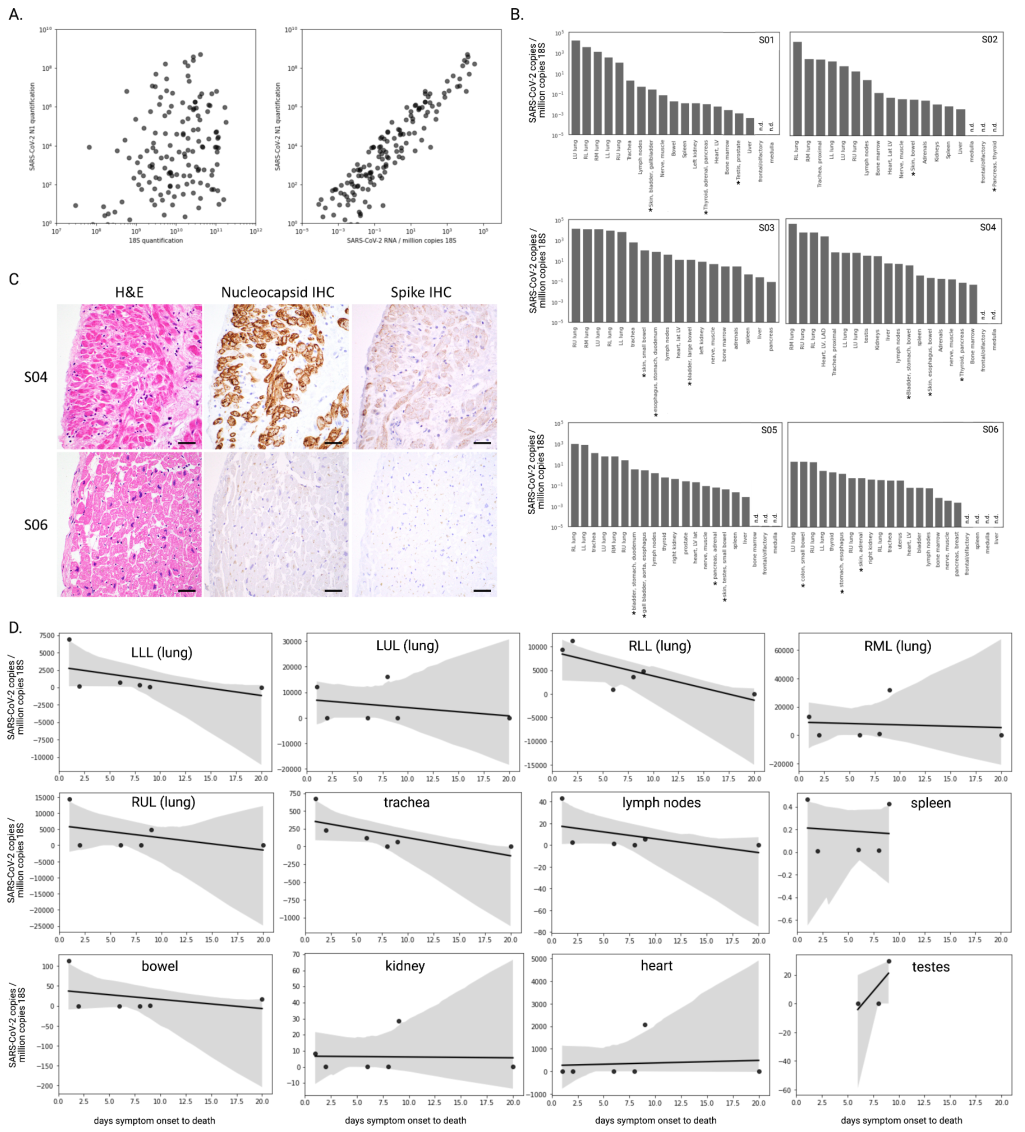

Supplementary Fig. 1: Viral loads across the sample set. a SARS-CoV-2 N1 quantification versus human ribosomal RNA 18S quantification, and SARS-CoV-2 N1 quantification versus normalized viral load. **b** Ordered viral load for all tissues within each subject, without summarization. Asterisk denotes a composite tissue. 'n.d.' denotes a SARS-CoV-2 N1 quantification below our limit of detection. **c** SARS-CoV-2 immunohistochemistry of heart. Sections of heart from S04 (top) show positive SARS-CoV-2 nucleocapsid (middle) and spike protein (right) immunostaining in cardiomyocytes in a predominantly subendocardial distribution, while sections from S06 (bottom) are negative for viral antigens. All images taken with 40x objective. Staining was performed once. Scale bars are 20-microns. **d** Normalized viral loads versus time between symptom onset and death for a selection of tissues. Lines represent a linear regression with a 95% confidence interval (shaded area).

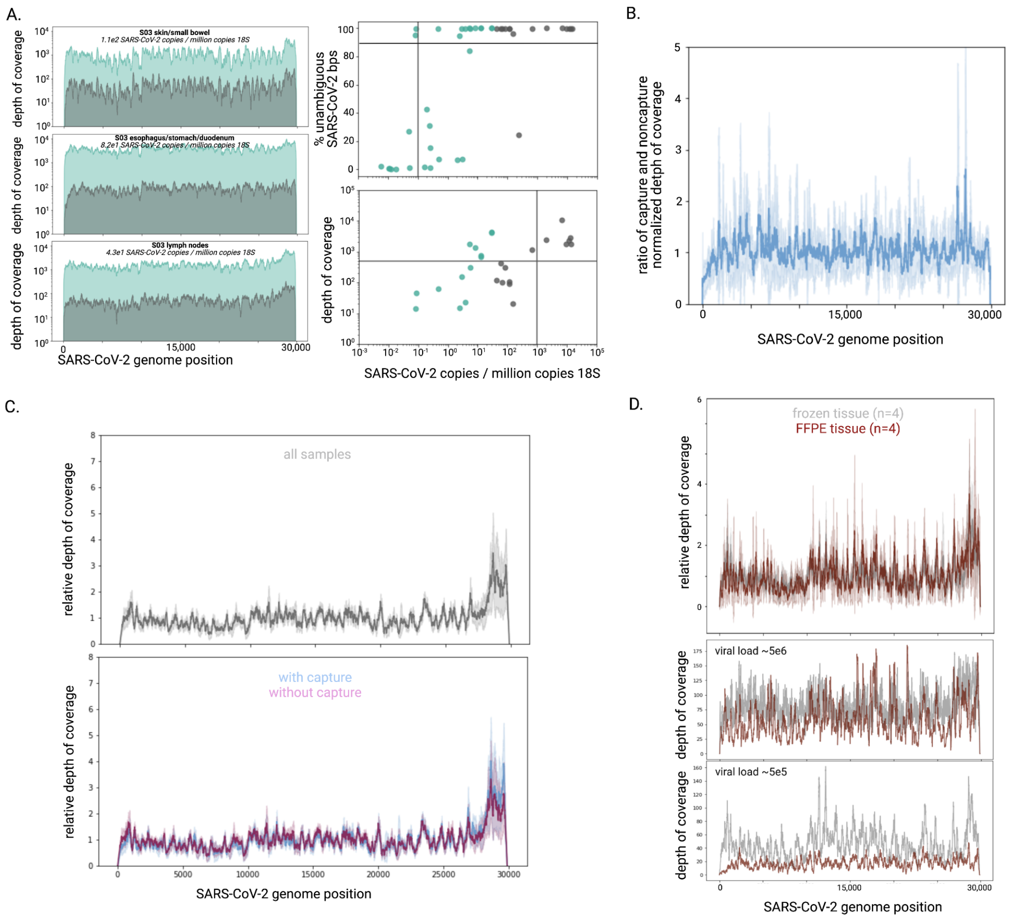

Supplementary Fig. 2: Viral sequencing with hybrid capture. a Selected sample libraries that were sequenced with and without hybrid capture (sequencing reads for each condition were downsampled to the same number of raw reads) and depth of genome coverage was calculated across the genome; specific viral load and sample are noted (left). A selection of pilot samples were sequenced and unambiguous assembly length and depth of coverage were calculated across the set (right; Supplementary Table 3, Methods). These data were used to determine normalized viral load thresholds for samples sequenced (normalized viral load >0.1), with (normalized viral load <1000), and without (normalized viral load >1000) hybrid capture. Samples sequenced with hybrid capture are represented with green and samples sequenced without hybrid capture are represented in grey. **b** For 12 libraries sequenced with and without hybrid capture, downsampled to the same number of raw reads, normalized viral loads were obtained across the SARS-CoV-2 genome. At each position in the genome, the ratio of normalized viral load in the condition sequenced with capture and without capture was calculated. The blue line represents the mean of that ratio across the 12 samples, and the light blue shadow represents the standard deviation. **c** Relative depth of coverage for all samples that yielded high-depth genomes (>500x mean depth of coverage). Grey line indicates mean relative depth of coverage across all samples, while shadow indicates standard deviation (top). Blue (with capture) and purple (without capture) lines represent mean relative depth of coverage across samples, while shadow indicates standard deviation (bottom). **d** Four FFPE (red) and four frozen (grey) tissue samples of approximately similar viral loads, downsampled to the same number of raw reads, were compared. Relative depth of coverage was calculated in each sample and condition, and the lines represent the mean of the ratio across all samples while the shadow represents standard deviation (top). Raw depth of coverage is shown for two representative comparisons of FFPE and frozen tissues (bottom).

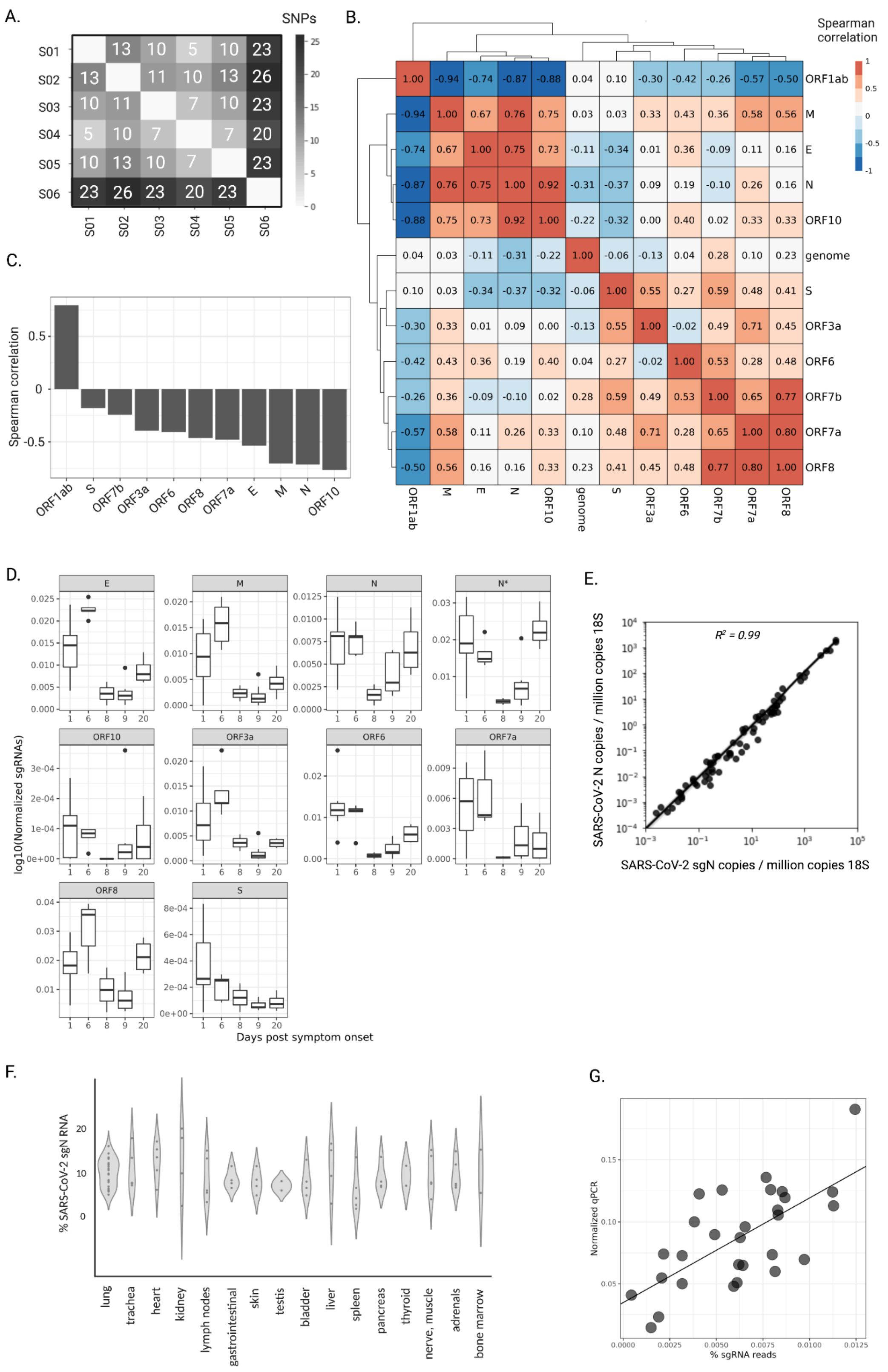

Supplementary Fig. 3: Comparison of viral genomes and subgenomic RNAs across and within subjects. a A distance matrix displayed SNPs between SARS-CoV-2 consensus genomes from each

subject (using the more common genome from S02); number of SNPs differentiating subjects is annotated. **b** A clustered heatmap of Spearman correlations in viral gene expression shows anti-correlation between ORF1ab and genes M, E, N, and ORF10. **c** Spearman correlation between individual viral genes and time post symptom onset. **d** sgRNA quantification by gene; boxes delineate quartiles and whiskers show the range, excluding outliers (as determined by the interquartile range). **e** sgN viral load vs N viral load reveals strong correlation (R^2=0.99). **f** Percent of sgRNA, or the ratio of sgN and N quantifications, grouped by tissues. **g** Scatterplot of qPCR vs Antenna ngs-based sgRNA quantification for the N gene sgRNA demonstrates agreement between the two approaches.

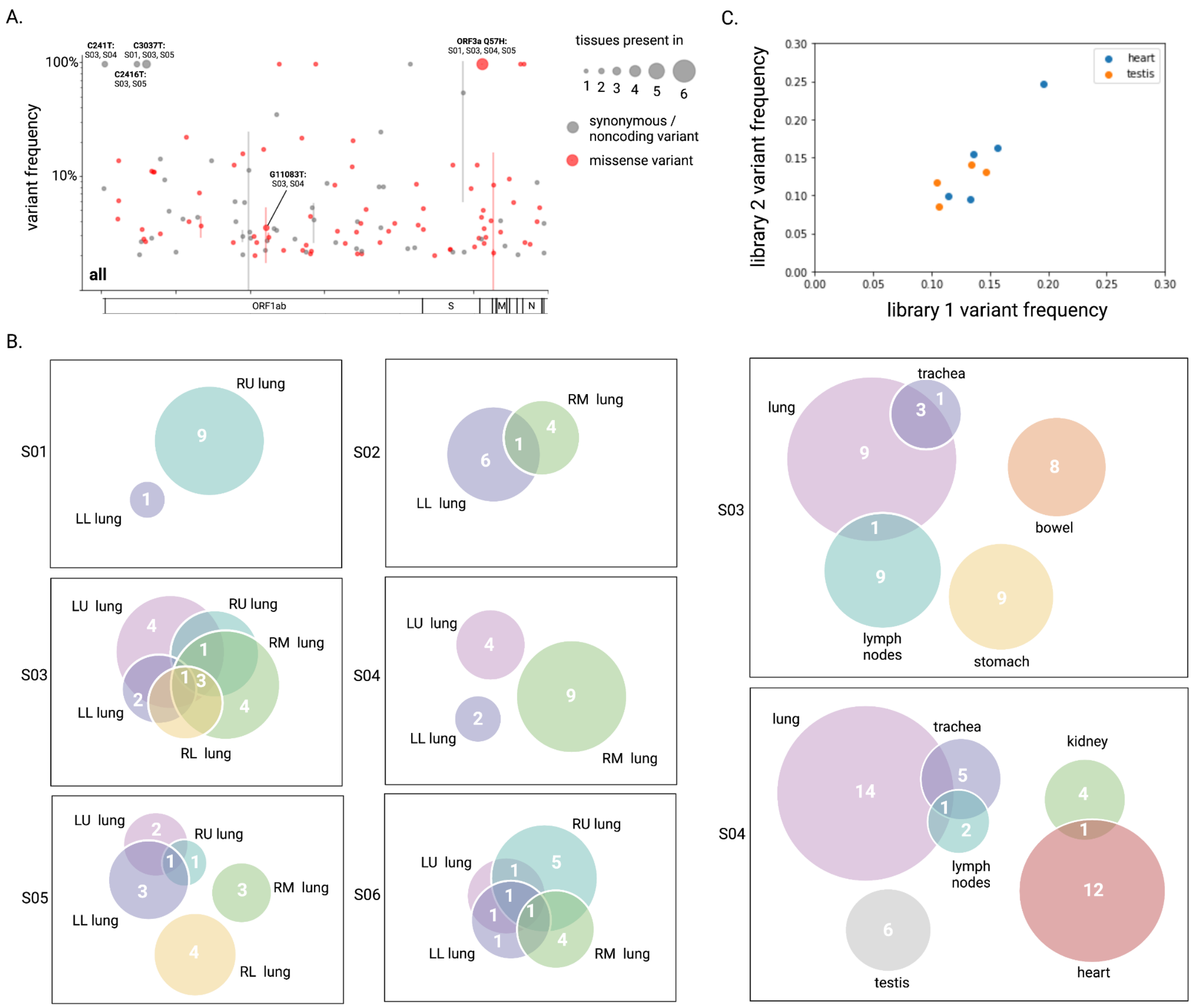

Supplementary Fig. 4: Minor viral variants of interest. a All variants identified across the entire sample set are displayed as variant frequency versus genome position. Point diameter reflects the number of subjects a variant occurred in. The standard deviation of the frequency at which the variant occurred across subjects or tissues is depicted by vertical bars. Red points reflect nonsynonymous changes whereas grey points reflect synonymous or noncoding variants. **b** Visualization of variants shared among lung tissues in S01-S06 (left) and among lung (combined across lung sections) and extrapulmonary tissues in S03 and S04 (right). Number of total variants in each tissue, and variants shared between tissues are noted. **c** Concordance of variant frequencies in high-frequency variants from S04. Two independent high-depth genomes were assembled for the heart and testis from this subject (only one library from the kidney had a high-depth genome, so we did not include the high-frequency variant from that tissue in this plot), we quantified variant frequency in each library separately, and compared the frequencies between the two.
