# Supplementary material for: High-depth sequencing characterization of viral dynamics across tissues in fatal COVID-19 reveals compartmentalized infection
Source: Nat Commun. 2023 Feb 2;14:574. doi: 10.1038/s41467-022-34256-y (PMC9894515; doi:10.1038/s41467-022-34256-y)
Supplement: Supplementary file 2 — Description of Additional Supplementary Files [file 41467_2022_34256_MOESM2_ESM.pdf]

Supplementary Data File 1: RT-qPCR data across the complete sample set. This includes a Ct and quantification (corrected for assay dilution factor; see Methods) for both human rRNA (18S) and SARS-CoV-2 (nucleocapsid RNA) for all samples. Samples where SARS-CoV-2 was not detected, or was detected at a quantification below the assay's reported limit of detection (5 copies/µL) are shaded in gray. Additionally included are Ct and quantification (corrected for dilution factor) for SARS-CoV-2 subgenomic RNA (sgN) for a subset of samples. Blank entries indicate that the assay was not performed on the sample.

Supplementary Data File 2: SARS-CoV-2 IHC details. Symbols: "+" is Positive; "−" is Negative; N/A: is data not available

Supplementary Data File 3: Sequencing statistics for a pilot set of samples used to determine viral load cutoffs for sequencing and hybrid capture. These data are presented visually in Supplementary Fig. 2a.

Supplementary Data File 4: Sequencing data across all samples sequenced. This includes basic sequencing statistics such as reads sequenced, reads aligning to SARS-CoV-2, unambiguous SARS-CoV-2 genome assembly length, and mean depth of coverage. Summary statistics that identify whether a complete genome was assembled (>90%), and whether it was considered high-depth of coverage (>500x) are also included. The asterisk denotes that this sample was subsequently removed from analyses (Methods).

Supplementary Data File 5: GISAID genome acknowledgments table.
