# Supplementary material for: High-depth sequencing characterization of viral dynamics across tissues in fatal COVID-19 reveals compartmentalized infection
Source: Nat Commun. 2023 Feb 2;14:574. doi: 10.1038/s41467-022-34256-y (PMC9894515; doi:10.1038/s41467-022-34256-y)
Supplement: Supplementary file 8 — Reporting Summary [file 41467_2022_34256_MOESM8_ESM.pdf]

nature portfolio

Corresponding author(s): Erica Normandin, Isaac Solomon, Katie Siddle

Last updated by author(s): Oct 7, 2022

Reporting Summary

Nature Portfolio wishes to improve the reproducibility of the work that we publish. This form provides structure for consistency and transparency in reporting. For further information on Nature Portfolio policies, see our Editorial Policies and the Editorial Policy Checklist.

Statistics

For all statistical analyses, confirm that the following items are present in the figure legend, table legend, main text, or Methods section.

n/a	Confirmed	
☐	☒	The exact sample size (*n*) for each experimental group/condition, given as a discrete number and unit of measurement
☐	☒	A statement on whether measurements were taken from distinct samples or whether the same sample was measured repeatedly
☒	☐	The statistical test(s) used AND whether they are one- or two-sided<br>*Only common tests should be described solely by name; describe more complex techniques in the Methods section.*
☒	☐	A description of all covariates tested
☒	☐	A description of any assumptions or corrections, such as tests of normality and adjustment for multiple comparisons
☐	☒	A full description of the statistical parameters including central tendency (e.g. means) or other basic estimates (e.g. regression coefficient) AND variation (e.g. standard deviation) or associated estimates of uncertainty (e.g. confidence intervals)
☐	☒	For null hypothesis testing, the test statistic (e.g. *F*, *t*, *r*) with confidence intervals, effect sizes, degrees of freedom and *P* value noted<br>*Give P values as exact values whenever suitable.*
☒	☐	For Bayesian analysis, information on the choice of priors and Markov chain Monte Carlo settings
☒	☐	For hierarchical and complex designs, identification of the appropriate level for tests and full reporting of outcomes
☐	☒	Estimates of effect sizes (e.g. Cohen's *d*, Pearson's *r*), indicating how they were calculated

Our web collection on statistics for biologists contains articles on many of the points above.

Software and code

Policy information about availability of computer code

Data collection	Quantitative PCR data was generated using Quant Studio 6 Flex Real-Time PCR System Software. Sequencing data was generated on Illumina NovaSeq SP machines.
Data analysis	Viral genomic analyses were performed through use of viral-ngs (v2.0.21) pipelines (dockstore.org/organizations/BroadInstitute/collections/pgs), as implemented on Terra platform (app.terra.bio). Genome alignments were performed using MUSCLE (DOI: 10.1093/nar/gkh340) implemented in Geneious Prime (v2021.1.1), and mafft (version 7.487). Viral strains classification was performed by search against using the Usher web interface. LoFreq (version 2) was used to identify minor viral variants. Biopython (1.79) was used to annotate the viral genome. The Antenna pipeline for viral gene and sgRNA quantification is available at https://github.com/broadinstitute/antenna and can be cited by DOI: 10.5281/zenodo.7182211.

For manuscripts utilizing custom algorithms or software that are central to the research but not yet described in published literature, software must be made available to editors and reviewers. We strongly encourage code deposition in a community repository (e.g. GitHub). See the Nature Portfolio guidelines for submitting code & software for further information.

Data

Policy information about availability of data

All manuscripts must include a data availability statement. This statement should provide the following information, where applicable:

- Accession codes, unique identifiers, or web links for publicly available datasets
- A description of any restrictions on data availability
- For clinical datasets or third party data, please ensure that the statement adheres to our policy

The sequencing data generated in this study have been deposited on NCBI under the accession code PRJNA720544. Other data are available upon request.

Field-specific reporting

Please select the one below that is the best fit for your research. If you are not sure, read the appropriate sections before making your selection.

- [x] Life sciences
- [ ] Behavioural & social sciences
- [ ] Ecological, evolutionary & environmental sciences

For a reference copy of the document with all sections, see nature.com/documents/nr-reporting-summary-flat.pdf

Life sciences study design

All studies must disclose on these points even when the disclosure is negative.

Sample size	No sample size calculation was performed. We selected six subjects with evidence of high viral loads and extrapulmonary pathology. For each of these six subjects, we profiled all tissue specimens available. Prior to this study, it was unknown whether differences in virus genomes would be detectable at all within individuals. We selected 6 individuals with higher viral loads in lungs to test this hypothesis, which was confirmed in the results of this study.
Data exclusions	We did not report minor variants in samples with <500x mean depth of viral coverage. We also excluded minor variants from one tissue specimen that had strong evidence of contamination with another sample in the sample set.
Replication	No statistical method was used to predetermine cohort sample size. Subjects were a convenience sample composed of individuals who had an autopsy performed at BWH following SARS-CoV-2 infection; blinding and randomization were not relevant to this study. Each FFPE tissue block was stained once with the respective SARS-CoV-2 nucleocapsid or spike antibodies; these antibodies have been extensively validated for diagnostic use in our clinical IHC laboratory, including reproducibility between staining batches. RT-qPCR experiments were performed in triplicate; mean quantifications are reported. Sequencing was performed and analyzed in duplicate, where possible. Variant profiles from samples with low mean viral coverage (<500x) were excluded from analyses, consistent with the threshold determined in this study (Figure 2).
Randomization	Randomization was not relevant in this study. All samples were treated identically.
Blinding	Blinding was not relevant to the study in which all subjects were known to be SARS-CoV-2 infected, and the goal was to detect differences in sequences between organs.

Reporting for specific materials, systems and methods

We require information from authors about some types of materials, experimental systems and methods used in many studies. Here, indicate whether each material, system or method listed is relevant to your study. If you are not sure if a list item applies to your research, read the appropriate section before selecting a response.

Materials & experimental systems

n/a	Involved in the study	
☐	☒	Antibodies
☒	☐	Eukaryotic cell lines
☒	☐	Palaeontology and archaeology
☒	☐	Animals and other organisms
☐	☒	Human research participants
☒	☐	Clinical data
☒	☐	Dual use research of concern

Methods

n/a	Involved in the study	
☒	☐	ChIP-seq
☒	☐	Flow cytometry
☒	☐	MRI-based neuroimaging

Antibodies

Antibodies used	SARS-CoV-2 nucleocapsid immunohistochemistry was performed; the antibody used was a rabbit polyclonal antibody (NB100-56576; Novus Biologicals, Centennial, CO; 1:500 dilution). Additionally, IHC for the SARS-CoV-2 spike protein was performed using a mouse monoclonal antibody (GTX632604; GeneTex, Irving, CA; 1:1000 dilution).
Validation	These antibodies have been extensively validated for diagnostic use in our clinical IHC laboratory, including reproducibility between staining batches.

Human research participants

Policy information about studies involving human research participants

Population characteristics	Subjects all had fatal cases of COVID-19. Demographics, medical histories, and disease courses varied widely, detailed in depth in the manuscript. The cohort of six included two females and four males, ages ranged from 50-68 years old, three were white (not hispanic), two were white (hispanic), and one was black.
Recruitment	The initial cohort was determined by convenience (consecutive autopsies at Brigham and Women's Hospital) and the six subjects studied in depth were selected due to abnormal histopathology. Subjects were a convenience sample composed of individuals who had an autopsy performed at BWH following SARS-CoV-2 infection. All subjects were evaluated, and individuals with the highest viral loads in lung were selected for more in depth analysis in order to maximize the chances of detecting virus in other tissue sites, as detailed in manuscript text.
Ethics oversight	This study was approved by the Mass General Brigham Institutional Review Board under a protocol allowing for use of excess tissue not required for diagnosis that was collected during routine hospital autopsy examination (#2015P001388). The protocol waived the requirement for consent from subjects who participated in the study due to their deceased status and overall risk which was deemed minimal. Consent for the hospital autopsy was previously given by the decedents' next of kin or health care proxy per Massachusetts state law, with agreement that tissue retained by BWH could be used for IRB-approved research studies.

Note that full information on the approval of the study protocol must also be provided in the manuscript.
